# Basophil activation test has high reproducibility and is feasible in the clinical setting

**DOI:** 10.1111/pai.13870

**Published:** 2022-11-01

**Authors:** Hannah Jaumdally, Matthew Kwok, Zainab Jama, Rochelle Hesse‐Lamptey, Richard McKendry, Oliver Galvez, Yvonne Daniel, Alexandra F. Santos

**Affiliations:** ^1^ Department of Women and Children's Health (Pediatric Allergy), School of Life Course Sciences, Faculty of Life Sciences and Medicine King's College London London UK; ^2^ Peter Gorer Department of Immunobiology, School of Immunology and Microbial Sciences King's College London London UK; ^3^ Children's Allergy Service, Evelina London Children's Hospital Guy's and St Thomas' Hospital London UK; ^4^ Asthma UK Centre in Allergic Mechanisms of Asthma London UK; ^5^ Viapath, Special Haematology Laboratory London UK

**Keywords:** anaphylaxis, basophil activation test, CD203c, CD63, diagnosis, food allergy, oral food challenge, peanut allergy

## Abstract

**Background:**

The basophil activation test (BAT) has high accuracy to diagnose peanut allergy and can reduce the need for oral food challenges (OFC); however, so far it has not been incorporated in clinical practice.

**Methods:**

We assessed the reproducibility of BAT within the same laboratory and between two different laboratories and the feasibility of using BAT in the clinical setting.

**Results:**

One hundred and two children being assessed for peanut allergy were tested on BAT (72 allergic, 30 sensitized tolerant). There was little internal variation (coefficient of variation <15%) in the BAT and a very strong correlation (*R*_s_ > .95) between BAT performed across laboratories. The 2 BAT methods were strongly correlated but not interchangeable. In the cases of discrepancy, our in house BAT method was 100% accurate. BAT was feasible and well‐accepted by clinicians: no patient with positive BAT was referred for OFC, leading to reduction in the number of OFC required. Twenty one percent of patients who underwent OFC reacted to peanut. A negative BAT also encouraged the performance of OFC in sensitized children who would otherwise be considered allergic, 50% of whom did not react and incorporated peanut in the diet.

**Conclusions:**

The BAT is a robust test that can reliably be transferred between laboratories; however, different BAT methods are not interchangeable. BAT was well integrated in the clinical decision‐making process in a specialized center.

## Graphical abstract

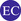

**Abbreviations**
BAT: basophil activation test
CV: coefficient of variation
fMLP: formyl‐methionyl‐leucylphenylalanine
MFI: mean fluorescence intensity
OFC: oral food challenge
RPMI: Roswell Park Memorial Institute medium
SPT: skin prick test
S/N: signal to noise ratio

**Key Message**

The Basophil Activation Test (BAT) can have consistent and reproducible results if the same methodology and standardization of cytometers is applied. In this single center study, it was feasible to perform the BAT in a cohort of patients seen in clinical practice. This level of standardization and confirmation of feasibility are crucial for future regulatory approval and successful transition to the clinic.

## 1 INTRODUCTION

Food allergy has become increasingly prevalent and severe in the recent decades, giving rise to increased awareness and increased need for testing.^1^, ^2^ In many cases, exposure to the allergen in a medically supervised and controlled environment in hospital during oral food challenge (OFC) is required to ascertain whether the child is allergic or not. However, OFC are resource‐intensive and place the patient at risk of potentially severe allergic reactions and the need for OFC far exceeds current capacity of Allergy services.

We and others have previously demonstrated that the basophil activation test (BAT) has high specificity and sensitivity to diagnose food allergy.^3^, ^4^, ^5^, ^6^, ^7^, ^8^, ^9^ For instance, for peanut allergy, BAT was accurate in 97% of cases and reduced the need for OFC in approximately 67%.^10^ We confirmed the diagnostic performance of BAT to peanut in a large well‐characterized cohort of children who participated in LEAP and associated studies.^11^ Bringing BAT to clinic would enhance the accuracy and safety of food allergy diagnosis. However, BAT is still a research test not available to clinicians seeing patients with suspected food allergy in the majority of clinical settings. Different stages need to be achieved for the transition of BAT to the clinic,^3^ including: 1. standardization of the methodology and reliability of its application in different laboratories; 2. technical validation and clinical validation of BAT; and 3. feasibility, for instance in terms of access to flow cytometry, transportation, and timely processing of samples.

In this study, we aimed to assess the consistency and reliability of BAT within the same laboratory and between two different laboratories and to assess the feasibility and acceptability of using BAT in the clinical setting.

## 2 METHODS

### 2.1 Study population

Two groups of subjects were tested in this study: adults with no known allergic diseases as healthy controls and children aged 6 months to 15 years being assessed for possible peanut allergy, that is, either had a history of reaction or unknown consumption of peanut and/or sensitization to peanut. Healthy adults were recruited following ethical approval (reference 14/LO/1699). Their samples were used for optimization and determination of intra‐assay variability, all the other results were generated with samples collected from children with suspected peanut allergy who were recruited between 2019 and 2021 as part of the study “Diagnostic markers of clinical allergy versus sensitization to peanut” (10/H0802/044), as previously described.^10^ The samples collected from healthy adult donors were stimulated with anti‐IgE, fMLP, and buffer alone. The samples collected from children were stimulated with peanut extract in different concentrations, in additions to anti‐IgE, fMLP, and buffer alone, as controls. Children underwent diagnostic assessment for peanut allergy, including clinical assessment, skin prick test (SPT), blood collection for specific IgE and BAT, and OFC if clinically indicated. Ethics approval was obtained and informed consent from adults with parental responsibility and assent from children were obtained prior to any study procedures.

### 2.2 Basophil activation test

The samples collected from healthy adult donors were used for technical validation of the assay and basophils were stimulated with anti‐IgE, fMLP, and buffer alone. In the samples collected form children, basophils were stimulated with peanut extract in different concentrations, in additions to anti‐IgE, fMLP, and buffer alone.

We have used two different methodologies for the basophil activation test: an in‐house method previously validated for peanut allergy^10^ and a method customized for our lab by Beckman Coulter with dry‐freezed antibodies and the same peanut extract (ALK‐Abello). These two methods are designated IH‐BAT and BC‐BAT, respectively, throughout the manuscript. BAT was performed within 4 h of blood collection for both methods. The two BAT methods were performed in parallel and across two laboratories, that is, the two BAT methods were tested on the same day using the same blood sample in two different laboratories: the Santos Lab at King's College London (KCL) and the Special Hematology Lab of Viapath, UK (DxLab). Flow cytometry was performed at each respective laboratory.

The IH‐BAT was performed as in previous studies.^10^, ^11^, ^12^ Briefly, 100 μl of heparinized whole blood was stimulated for 30 min at 37°C and 5% CO_2_ with two optimal concentrations of peanut extract (ALK, Abello) 10 and 100 ng/ml,^10^ alongside a negative control containing RPMI alone (ThermoFisher), and two positive controls: polyclonal goat anti‐human IgE antibody control (1 μg/ml; Sigma‐Aldrich), and formyl‐methionyl‐leucylphenylalanine (fMLP, 1 μM; Sigma‐Aldrich). Following stimulation, the basophils were stained with the following antibodies: CD123‐FITC, CD203c‐PE, HLADR‐PerCP, and CD63‐APC (Biolegend) at 4°C for 30 min. Prior to erythrocyte lysis with BD Pharm Lyse (BD Biosciences), excess and unbound antibodies were washed off using staining buffer (PBS with 2 mM EDTA and 0.5% Bovine Serum Albumin) followed by centrifugation at 300 *g* for 5 min at 4°C. Flow cytometry was performed using a CytoFLEX (Beckman Coulter) or BD Canto II, and the results were analyzed using FlowJo software (version 10.6.2; Ashland).

For the BC‐BAT, 50 μl of Dulbecco's PBS (pH 7.2) were initially added to reconstitute the dry‐freezed antibodies (CD45‐KO, CD3‐PC7, CRTH2 APC, CD203c PE, CD63 PB450 from Beckman Coulter) containing peanut extract (ALK‐Abello) at serial 10‐fold dilutions from 10 μg/ml to 0.1 ng/ml or anti‐IgE or fMLP provided by BC, as controls. This was then followed by the addition of 50 μl of heparinized whole blood, and incubation at 37°C and 5% CO_2_ for 20 min. An erythrolytic reagent, OptiLyse C (Beckman Coulter) was then added, and any excess and unbound antibodies were washed off with Dulbecco's PBS followed by a final centrifugation at 300 *g* for 5 min at RT. Flow cytometry was performed using CytoFLEX (Beckman Coulter), and data were analyzed using FlowJo software (version 10.6.2; Ashland). Figure S1 shows the gating strategies adopted for both BAT methods. Non‐responders were defined by a %CD63+ Basophils following stimulation with anti‐IgE of <5% and with fMLP of 5% or more.

### 2.3 Standardization of flow cytometers

To reduce variability between the two Cytoflex platforms, we first optimized the gain settings of the cytometers. A gain titration was performed on the first Cytoflex to determine the optimal gain for each channel on the KCL Cytoflex using 8 peak rainbow calibration particles (P‐RCP8‐3.0; Kisker Biotech). This involved setting the FITC gain to 400 and adjusting all other fluorescence gains to 10 before recording 5000 bead events. Next, all fluorescence gains except FITC were increased to 20 and recorded as previously. This step was repeated by increasing the gain by 10 each time until reaching 100, then increasing the gain by 100 until reaching 1000, and finally increasing the gain by 250 until reaching the maximum gain of 3000. Subsequently, the median and standard deviation of the 1st, 2nd, and 4th peak for each channel was extracted. The same method was then performed by setting the PE gain to 200, and adjusting all other fluorescence gains starting at 10 and repeating the entire procedure as described for the FITC gain titration.

For determination of minimum gains, the signal to noise ratio (S/N) defined as MFI (peak 2)/MFI (peak 1) was calculated and plotted as a function of gain. Additionally, the coefficient of variation (CV) of peak 4 was also plotted as a function of gain. To determine the minimum gain for each channel, the point at which the S/N and rCV plots plateau was selected as the minimum gain value. The final optimal gain value for each channel was then calculated as an average of the minimum gain values selected in the previous step.

Standardization was performed between the two Cytoflex platforms following the manufacturer's instructions (version B49006AP) using the optimal values selected above. Target median values were generated using Daily QC fluorospheres (B53230; Beckman Coulter) from a specific lot for each channel on the first Cytoflex. Resulting median fluorescence intensities were calculated from a total of six replicates and used as the standardization target value. The average MFI values were then matched as close as possible on the second Cytoflex using the same lot of QC fluorospheres to create a reference standardization file. Standardization was performed using the same lot of beads before each experiment on each Cytoflex.

### 2.4 Statistical analyses

The distribution of variables was not normal as assessed by the Kolmogorov–Smirnov and Shapiro–Wilk tests, thus non‐parametric tests were used. Mann–Whitney U test and Wilcoxon rank were used for comparison of the distribution of quantitative variables between independent and paired groups, respectively. Spearman correlation and Bland–Altman plots were used to assess the relationship between paired variables, namely the comparison of BAT results using two different methods tested across two different laboratories. We expected that the CV of the BAT was ≤15% and that the correlation of results obtained across methods and across laboratories was ≥0.90. ROC curve analyses were used to determined the diagnostic utility of the tests. The peanut concentration with the largest area under the ROC curve for each method was selected and the optimal cut‐off defined by the Youden index was selected for the optimal concentration for each method. Most analyses were performed with SPSS 27.0 (IBM Inc) and graphs were designed using GraphPad Prism 9.0 (GraphPad Software, Inc).

## 3 RESULTS

### 3.1 The basophil activation test show little internal variation

We assessed the variability and reproducibility of BAT by testing the same conditions repeatedly (*n* = 10) by two different operators. The coefficient of variation ± standard deviation was 3.48 ± 1.92% and 10.32 ± 3.69% for IH‐BAT in the hands of a more and a less experienced operators, respectively (Figure S2). We also compared the IH‐BAT analyzed in two different cytometers (BD Canto II versus BC Cytoflex) in parallel and observed a high consistency in the results obtained, particularly if the cytometers were standardized before analyses (data not shown).

### 3.2 The basophil activation test has high reproducibility when tested in separate laboratories

**FIGURE 1:**
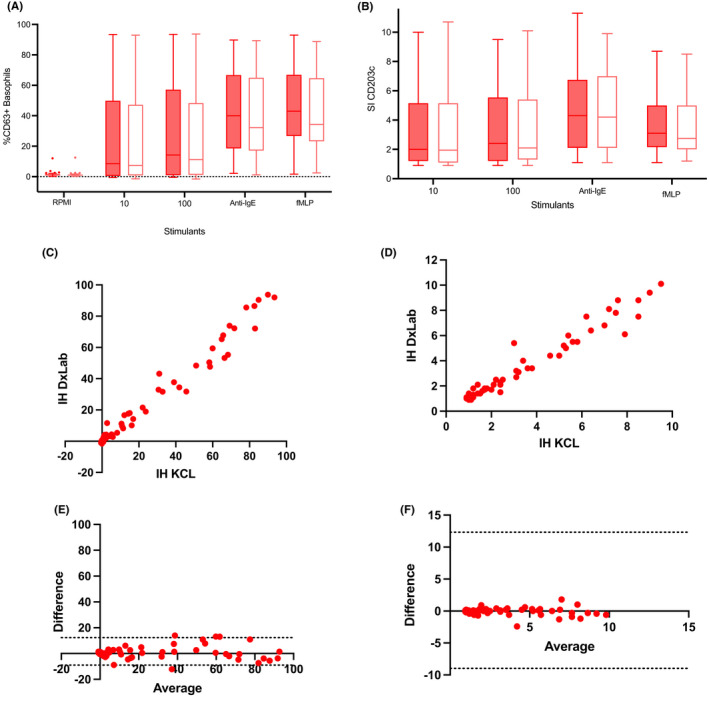
Head‐to‐head comparison between the in‐house basophil activation test (IH‐BAT) across laboratories, a research laboratory (KCL) and a diagnostic laboratory (DxLab) in terms of: basophil activation using CD63 (A) and CD203c (B) in a variety of stimulating conditions (RPMI alone, peanut extract 10 and 100 ng/ml, anti‐IgE, and fMLP); correlation of CD63+ basophils (C) and stimulation index of CD203c (D) following stimulation with 100 ng/ml of peanut extract; Bland–Altman plot of difference over average of basophil activation following stimulation with 100ng/ml of peanut extract using CD63 (E) and CD203c (F).

We recruited 102 children being assessed for possible peanut allergy (Table 1). We performed the IH‐BAT in two different laboratories, one research laboratory, and one clinical diagnostic laboratory, using 65 samples from these children assessed for possible peanut allergy. The IHBAT results obtained across laboratories were comparable (Table 2, Figure 1). The correlation of the results obtained between the two laboratories was very strong, with correlation coefficients above .95 for all allergen concentrations tested, and the Bland–Altman bias was very low. A second BAT method (BC‐BAT) tested in parallel (*n* = 65) showed statistically significant differences between laboratories in some conditions, despite the strong correlation, both using CD63 and CD203c activation markers (Table S1, Figure S2).

**TABLE 1:** Characteristics of study population. Data are presented as number and percentage for qualitative variables or median and inter‐quartile range for quantitative variables.

Characteristics	Overall (*N* = 102)	Peanut‐Allergic (*n* = 72)	Peanut‐sensitized non‐allergic (*n* = 30)	*p* value*
Age (years)	2.7 (1.1; 7.0)	3.1 (1.2; 7.3)	2.2 (0.9; 6.1)	.201
Males	63 (62%)	43 (60%)	20 (67%)	.655
History of reaction to peanut	48 (48%)	38 (54%)	10 (33%)	.082
Atopic dermatitis	48 (47%)	32 (44%)	16 (53%)	.515
Other food allergies	60 (59%)	45 (63%)	15 (50%)	.171
Asthma/wheeze	13 (13%)	10 (14%)	3 (10%)	.751
Allergic rhinitis	29 (28%)	21 (29%)	8 (27%)	1.0
Equivocal diagnosis at referral (before BAT)	85 (83%)	55 (76%)	30 (100%)	**.002**
Skin prick test (mm)	4 (1; 8)	6 (3; 9)	0 (0; 3)	**<.001**
Specific IgE to peanut (KU_A_/L)	1.94 (0.35; 6.35)	3.79 (1.33; 16.63)	0.15 (0.10; 0.60)	**<.001**
Ara h 2‐sIgE (KUA/L)	0.24 (002; 3.90)	1.98 (0.12; 9.71)	0.01 (0.01; 0.12)	**<.001**
BAT to peanut (%CD63+ Basophils 10‐100 ng/ml of peanut extract)	10.1 (0.98; 40.35)	23.21 (9.30; 55.24)	0.50 (0; 2.26)	**<.001**

* *p* value is shown for the comparison between peanut allergic and peanut sensitized tolerant children using Fisher's Exact Test for qualitative variables and the Mann–Whitney U test for quantitative variables. The bold values indicates *p* < .05.

The bold values indicates *p* < .05.

**TABLE 2:** Comparison of results of the in‐house basophil activation test (IH‐BAT) across laboratories (*n* = 65)

BAT Parameters	KCL	DxLAB	Comparison of BAT across labs		
			Wilcoxon Test	Spearman correlation	Bland–Altman
%CD63 @10 ng/ml	10.2 (0.4; 49.2)	7.3 (0.8; 47.2)	*p* = .224	*R*_s_ = .973 *p* < .001	*B* = 1.674 ± 5.432
%CD63 @100 ng/ml	14.6 (0.9; 56.7)	11.2 (1.1; 48.4)	*p* = .367	*R*_s_ = .980 *p* < .001	*B* = 0.749 ± 4.923
SI CD203c @10 ng/ml	2.1 (1.2; 5.1)	2.0 (1.1; 5.1)	*p* = .527	*R*_s_ = .967 *p* < .001	*B* = 0.082 ± 0.581
SI CD203c @100 ng/ml	2.4 (1.2; 5.5)	2.1 (1.3; 5.4)	*p* = .497	*R*_s_ = .972 *p* < .001	B = −0.064 ± 0.563

Abbreviations: *B*, bias (mean and standard deviation); *R*_s_, Spearman correlation coefficient.

### 3.3 The results of the basophil activation test using two different methods are not interchangeable

**FIGURE 2:**
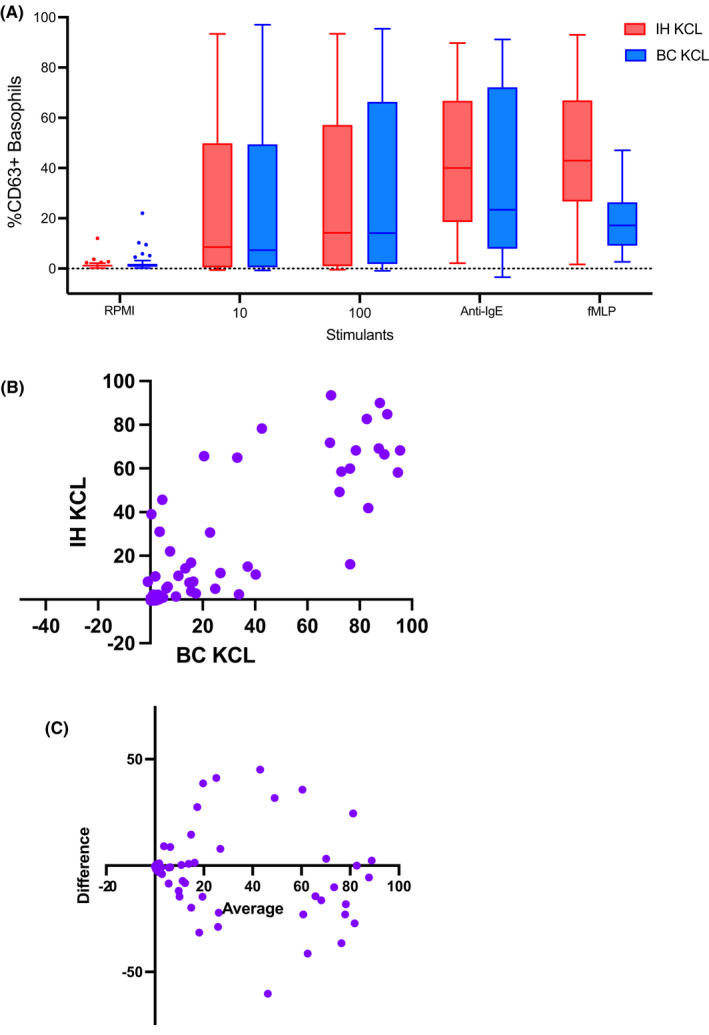
Comparison of basophil activation measured with CD63 using two BAT methods performed at the KCL Lab: IH, in house‐BAT method and BC, Beckman Coulter method.

We compared two different BAT methods head‐to‐head (IH‐BAT versus BC‐BAT) using samples of children assessed for possible peanut allergy (*n* = 102). Although the proportion of CD63‐positive basophils following peanut stimulation was not significantly different between the two methods, the MFI for the activation marker CD203c was lower using the BC‐BAT compared with IH‐BAT in all conditions tested (Table 3, Figures 2 and S3). The correlation between the basophil activation using CD63 measured with the two BAT methods was strong and the bias calculated with Bland–Altman was low; however, the dispersion of the results was substantial. The correlation of results obtained using the second activation marker CD203c was also strong but the levels of CD203c expression measured with the BC‐BAT method were systematically lower than the levels measured with the IH‐BAT method.

**TABLE 3:** Comparison of two different methods for the basophil activation test performed in the KCL laboratory (*n* = 102). Basophil activation test: mechanisms and considerations for use in clinical trials and clinical practice

BAT Parameters	IH‐BAT	BC‐BAT	Comparison of 2 BAT methods		
			Wilcoxon Test	Spearman correlation	Bland–Altman
%CD63 @10 ng/ml	10.2 (0.4; 49.2)	5.0 (0.4; 33.0)	*p* = .316	*R*_s_ = .835 *p* < .001	*B* = −0.140 ± 11.16
%CD63 @100 ng/ml	14.6 (0.9; 56.7)	6.6 (1.3; 37.2)	*p* = .077	*R*_s_ = .779 *p* < .001	*B* = −3.020 ± 19.36
SI CD203c @10 ng/ml	2.1 (1.2; 5.1)	1.3 (1.0;2.0)	***p* < .001**	*R*_s_ = .809 *p* < 0.001	*B* = 1.40 ± 1.783
SI CD203c @100 ng/ml	2.4 (1.2; 5.5)	1.3 (1.1; 2.3)	***p* < .001**	*R*_s_ = 0.790 *p* < 0.001	*B* = 1.323 ± 1.771
Number of basophils @10 ng/ml	1526 (931; 1958)	836 (513; 1147)	***p* < .001**	*R*_s_ = .753 *p* < .001	*B* = 610.8 ± 393.9
Number of basophils @100 ng/ml	1546 (865; 1936)	802 (513; 1164)	***p* < .001**	*R*_s_ = .674 *p* < 0.001	*B* = 623.7 ± 427.9
% Basophils @10 ng/ml	0.32 (0.23; 0.45)	0.32 (0.21; 0.44)	***p* < .001**	*R*_s_ = .825 *p* < .001	*B* = 0.069 ± 0.121
% Basophils @100 ng/ml	0.33 (0.22; 0.47)	0.31 (0.20; 0.43)	***p* < .001**	*R*_s_ = .823 *p* < .001	*B* = 0.082 ± 0.123

The bold values indicates *p* < .05.

There did not seem to be a systematic error between the methods; however, at the individual patient level, a discordance between the results obtained with the two methods was evident. For instance, patients tested negative with one method while testing positive with the other method, which can have diagnostic implications. Positivity of BAT results was defined for each method based on the optimal cut‐offs determined by the Youden index in ROC curve analyses (*n* = 95 for IH‐BAT and *n* = 82 for BC‐BAT, as participants with non‐responding basophils were excluded, Figure S5). Considering only the patients that underwent OFC (*n* = 32), there were four patients (out of 32, 12.5%) for whom the two BAT methods provided opposite results (Table 4). Out of these, IH‐BAT was correct in 4/4 (100%) of cases. The cases of misdiagnosis using BC‐BAT alone were as many false negatives as false positives. False negatives are the most concerning as they can result in accidental reactions in the community if the BAT was used in isolation, without OFC. Using our proposed approach of doing OFC in all patients with negative BAT, the false‐negative cases would not have resulted in accidental reactions in the community, but BC‐BAT (but not IH‐BAT) would still have resulted in two cases of overdiagnosis of peanut allergy.

**TABLE 4:** Comparison of outcomes for BAT results obtained with the 2 BAT methods in relation to allergic status as determined by oral food challenge (*n* = 32), highlighting the discrepancies between the two test results and its diagnostic implications

	Peanut allergic (by OFC)	Peanut‐sensitized tolerant (by OFC)	Total
IH‐BAT Positive BC‐BAT Positive	1	0	1
IH‐BAT Positive BC‐BAT Negative	2	0	2
IH‐BAT Negative BC‐BAT Positive	0	2	2
IH‐BAT Negative BC‐BAT Negative	4	23	27
Total	7	25	32

*Note*: Patients who did not undergo oral food challenge or had non‐responding basophils were excluded.

### 3.4 Integration of the basophil activation test in clinical decision‐making

In the last stage of the project, the results of BAT were fedback to the referring clinician for 79 participants, who were referred by 21 different healthcare professionals within our specialized center, with three main contributors, A.F.S, G.d.T. and T.M. (Table S4). All patients who had a positive BAT were not referred for OFC by 14 different clinicians (NB: the results of participants represented in Table 4 who underwent OFC despite positive BAT were not part of the last stage of the project and therefore not communicated to the clinicians). Among patients who did not have a positive BAT and underwent OFC, 21% were positive. Surprisingly, a negative BAT encouraged the clinicians to refer for OFC patients who had IgE sensitization with titers of IgE to peanut >0.10KU/L and <15 KU/L, 50% of whom passed the OFC and were able to incorporate peanut into their diet. Altogether, these data support the feasibility of using BAT in clinical practice and provide evidence of the acceptance of BAT among clinicians and of its integration in clinical decision‐making in a specialized center.

## 4 DISCUSSION

The BAT to peanut has previously shown high accuracy (97%) in the diagnosis of peanut allergy and ability to reduce the need for OFCs by two thirds^10^; however, it is yet to be part of routine clinical practice. Some barriers can be identified in the transition of BAT to the clinic, namely the need for standardization, technical and clinical validation, for overcoming logistical aspects and for clinical implementation. In this study, we demonstrated that it is possible to standardize the BAT methodology to a very high standard (CV < 15%), to obtain very consistent (*R*_s_ > .95) results across laboratories when the same BAT methodology is used, and the cytometer settings are standardized. However, different laboratory procedures produce different results, and this can have diagnostic implications. By ensuring the availability of flow cytometry for BAT with the use of dedicated bench‐top cytometers and using a courier for timely transportation of blood samples at room temperature, we were able to confirm the feasibility of testing BAT in a clinical diagnostic laboratory. Finally, BAT was well accepted by clinicians and integrated in clinical decision‐making, encouraged the performance of OFC in patients with detectable IgE and a negative BAT that would otherwise have been considered allergic and still reduced the overall number of OFC, in our center.

This study was funded by the MRC Confidence in Concept Scheme to address the barriers to the transition of BAT to the clinic. Equally important to asking probing questions at the bedside and addressing them with rigorous scientific methods is to facilitate the transition of the novel findings for the benefit of patients. A survey done by the EAACI Task Force for the Quality Assurance of BAT^13^ served as a proof‐of‐concept that it is possible to undertake round robins using blood samples sent from a central laboratory that various laboratories could test overtime for the purpose of quality assurance. As part of this exercise, however, it was evident that different BAT methodologies provided different results, despite the general overall agreement in terms of positive/negative result. This observation raised the question as to whether it is possible to standardize the methodology, both for the in vitro test and for the flow cytometry, and transfer the assay reliably between laboratories. The present study systematically compared BAT results across different methods, cytometers, and independent laboratories.

There was a clear difference in technical and diagnostic performance and in the consistency of results obtained across laboratories between the two BAT methods. For instance, the MFI for CD203c was consistently lower for BC‐BAT than for IH‐BAT. The differences in antibody clone, fluorochrome conjugate, and antibody/fluorochrome ratio could have contributed to this systematic difference. This difference in MFI obtained with the two methods had diagnostic implications, with the IH‐BAT having a higher area under the ROC curve for this parameter (0.957 versus 0.860 with BC‐BAT, see Figure S4). The fact that BC‐BAT performed differently across the two laboratories was surprising since the BC‐BAT protocol were performed in parallel, and it includes a fixative thus samples are meant to be stable for long periods. The comparison of methods within one laboratory and within laboratories was performed in a very similar way in terms of timings and flow of laboratory work. The high consistency obtained within methods between laboratories and the lower consistency between methods suggests that differences in consistency and consequently diagnostic performance is due to differences in methodology. For some parameters, the differences may be subtle at the population level judging by the statistical measures used; however, at the individual level to support the diagnosis of specific patients, the two BAT methods would potentially have led to opposite clinical decisions with major impact on patient outcomes. Taken the data altogether with special attention to the precision to diagnose individual patients, the IH‐BAT showed superior diagnostic performance, superior consistency across laboratories, lower number of non‐responders and lower number of spontaneous activation with the negative control. These observations led us to prefer the IH‐BAT method for future studies and clinical use to support the diagnosis of food allergy.

Interestingly, apart from the cases that had a positive BAT and were therefore dispensed of an OFC that would be otherwise positive, BAT also encouraged clinicians to refer for OFC children who had negative BAT despite detectable allergen‐sIgE. This underscores the point that BAT is unlikely to lead to an elimination of OFC and may instead create additional referrals. Furthermore, the potential overall reduction in the number of OFC performed would allow Allergy services to improve their capacity of response to demand and to perform OFC for indications other than diagnosis or resolution of food allergy. Adopting BAT for various food allergies could allow a more precise diagnosis and, consequently, shorter waiting times for OFC, allowing timely reintroduction of the suspected foods in the child's diet.

Although the present study focuses only on peanut allergy and the way diagnostic tests perform is allergen‐specific, it provides an important proof‐of‐concept for the implementation of BAT in clinical practice. A recent survey done as part of a task‐force of the EAACI indicated a high interest in the clinical application of the BAT and a high number of laboratories that have the necessary set up to offer this test.^13^ External quality assurance is needed to ensure the quality control of results and reliability of clinical tests. Round robins can be offered to laboratories to verify the system in place, such system of external quality assurance should however be specific to the BAT method in place.

From a practical standpoint, to laboratories looking to implement the BAT, strategies to overcome current barriers in transitioning the BAT into clinical practice include:

1. Adopting a specific BAT protocol and implement rigorous laboratory protocols and quality control measures;
2. Defining specific flow cytometer settings for the assay and ensure stringent standardization and calibration of the equipment;
3. Securing a dedicated cytometer to prevent prioritization of the equipment for testing for other conditions that can be considered more urgent;
4. Ensuring a system for timely transportation of blood to the laboratory and consider a system of patient booking.

Automated data analyses are another aspect to consider to reduce time and effort related to the flow cytometry analyses and to improve standardization and objectivity.^14^ Cost‐effectiveness studies of integrating BAT in the diagnostic work‐up for peanut and other food allergies and the education of health‐care professionals in the use and interpretation of BAT to support the diagnosis of food allergy are additional important aspects to facilitate the clinical application of BAT in the future.

For now, this proof‐of‐concept study demonstrated that BAT can have consistent and reproducible results if the same methodology and rigorous standardization are applied and that BAT is feasible and well‐accepted in the clinical setting. This level of standardization and confirmation of feasibility are crucial for future regulatory approval and successful transition to the clinic.

## FUNDING INFORMATION

This work was supported by the Medical Research Council (MRC Confidence in Concept Scheme MC/PC/18052, MRC Clinician Scientist Fellowship MR/M008517/1 and MRC Transition Support MR/T032081/1 awarded to A.F. Santos), Asthma UK (AUK‐BC‐2015‐01) and by the UK National Institute for Health Research comprehensive Biomedical Research Centre award to Guy's and St Thomas' National Health Service (NHS) Foundation Trust, in partnership with the King's College London and King's College Hospital NHS Foundation Trust.

## CONFLICT OF INTEREST

Dr. Santos reports grants from Medical Research Council (MR/M008517/1; MC/PC/18052; MR/T032081/1), Food Allergy Research and Education (FARE), the NIH, Asthma UK (AUK‐BC‐2015‐01) and the NIHR through the Biomedical Research Centre (BRC) award to Guy's and St Thomas' NHS Foundation Trust, during the conduct of the study; grants from Immune Tolerance Network/National Institute of Allergy and Infectious Diseases (NIAID, NIH); personal fees from Thermo Scientific, Nutricia, Infomed, Novartis, Allergy Therapeutics, Buhlmann, as well as research support from Buhlmann and Thermo Fisher Scientific through a collaboration agreement with King's College London. The other authors have nothing to disclose.

## Supporting information


Appendix S1
