# Supplementary material for: Basophil activation test has high reproducibility and is feasible in the clinical setting
Source: Pediatr Allergy Immunol. 2022 Nov 1;33(11):e13870. doi: 10.1111/pai.13870 (PMC9828203; doi:10.1111/pai.13870)
Supplement: Supplementary file 1 — Appendix S1 [file PAI-33-0-s001.docx]

**Online Supplementary material**

**The basophil activation test has high reproducibility and is feasible in the clinical setting**

Hannah Jaumdally MBBS^1,2,3^, Matthew Kwok BSc^1,2,4^, Zainab Jama BSc^1,2,4^, Rochelle Hesse-Lamptey MSc^2^, Richard McKendry PhD^1,2,4^, Oliver Galvez MSc^5^, Yvonne Daniel PhD^5^, Alexandra F. Santos, MD PhD^1,2,3,4*^

^1^Department of Women and Children’s Health (Pediatric Allergy), School of Life Course Sciences, Faculty of Life Sciences and Medicine, King’s College London, London, United Kingdom

^2^Peter Gorer Department of Immunobiology, School of Immunology and Microbial Sciences, King’s College London, London, United Kingdom

^3^Children’s Allergy Service, Evelina London Children’s Hospital, Guy’s and St Thomas’ Hospital, London, United Kingdom

^4^Asthma UK Centre in Allergic Mechanisms of Asthma, London, United Kingdom

^5^Viapath, Special Haematology Laboratory, London, United Kingdom

***Corresponding Author:**

Alexandra F. Santos

Address: Department of Paediatric Allergy, 2^nd^ floor, South Wing, St Thomas’ Hospital, SE1 7EH London, United Kingdom

Telephone number: +44 (0) 20 7188 6424

Fax number: +44 (0) 20 7403 8640

Email address: alexandra.santos@kcl.ac.uk

**E-Tables**

**Table E1.** Comparison of results of the customised basophil activation test (BC-BAT) across laboratories (n=6).

| **BAT Parameters** | **KCL** | **DxLAB** | **Comparison of BAT across labs** | | |
| --- | --- | --- | --- | --- | --- |
|  |  |  | **Wilcoxon Test** | **Spearman Correlation** | **Bland-Altman** |
| BC-%CD63  @10ng/ml | 5.0 (0.4; 33.0) | 7.2 (0.4; 53.4) | 0.258 | Rs=0.944  P<0.001 | B= 0.146 ± 4.915 |
| BC-%CD63  @100ng/ml | 6.6 (1.3; 37.2) | 11.5 (1.3; 59.2) | **0.010** | Rs=0.952  P<0.001 | B= 1.435 ± 5.586 |
| BC-SI CD203c  @10ng/ml | 1.3 (1.0 ;2.0) | 1.4 (1.1 ; 2.7) | **0.031** | Rs=0.934  P<0.001 | B= -0.068 ± 0.357 |
| BC-SI CD203c @100ng/ml | 1.3 (1.1 ; 2.3) | 1.6 (1.1 ; 2.8) | 0.273 | Rs=0.931  P<0.001 | B=-0.055 ± 0.351 |

**Table E2.** Comparison of two different methods for the basophil activation test performed in the KCL laboratory in terms of absence of response to IgE-mediated stimulants (non-responders) and high spontaneous activation in the negative control (High background)

| **BAT Parameters** | **IH-BAT** | **BC-BAT** |
| --- | --- | --- |
| Non-responders | 4% | 14% |
| High background | 4% | 9% |

**Table E3.** Diagnostic performance of two different methods for the basophil activation at the optimal concentrations of peanut extract (10 and 100 ng/ml) in all participants (n=98 for IH-BAT and n=82 for BC-BAT, non-responders were excluded).

| **BAT Parameters** | **AUC ROC** | **Optimal cut-off** | **Sensitivity (%)** | **Specificity (%)** | **PPV (%)** | **NPV (%)** |
| --- | --- | --- | --- | --- | --- | --- |
| IH-%CD63 @100ng/ml | 0.929 (0.879; 0.978) | 5.55% | 86 (75; 93) | 90 (73; 98) | 95 (87; 98) | 72 (59; 82) |
| IH-SI CD203c @100ng/ml | 0.957 (0.923;0.991) | 1.66 | 81 (70; 90) | 100 (88; 100) | 100 (100; 100) | 69 (71; 87) |
| BC-%CD63 @10ng/ml | 0.892 (0.819;0.966) | 8.29% | 75 (62; 86) | 92 (75; 99) | 96 (85; 99) | 63(52; 73) |
| BC-SI CD203c @10ng/ml | 0.895 (0.824 ; 0.965) | 1.15 | 82 (70; 91) | 89 (70; 98) | 94 (84; 98) | 70 (56; 80) |

**Table E4.** Diagnostic pathway for participants in the study and clinical decision making (n=79). Highlighted in bold are the study participants whose pathway was influenced by BAT.

| History | SPT / sIgE | Ara h 2 | BAT | OFC |
| --- | --- | --- | --- | --- |
| Reaction  N = 40 | >95%PPV n=19 | >1 KU/L  N=17 | **Positive N=15** | Not done N=15 |
|  |  |  | NR N=1 | Not done N=1**^a^** |
|  |  |  | Negative N=1 | Not done N=1**^a^** |
|  |  | <1 KU/L  N=2 | **Positive N=1** | Not done N=1 |
|  |  |  | **Negative N=1** | Positive N=1 |
|  | <95% PPV  >95% NPV  N=14 | >1 KU/L  N=3 | **Positive N=3** | Not done N=3 |
|  |  | <1 KU/L  N=11 | **Positive**  **N=6** | Not done N=6 |
|  |  |  | **Negative**  N=5 | **Positive N=2**  **Negative N=2**  Not done N=1**^b^** |
|  | <95% NPV  N=7 | <1 KU/L  N=7 | **Positive N=3** | Not done N=3 |
|  |  |  | **Negative**  **N=4** | Negative N=4 |
| Equivocal  N= 39 | >95%PPV  n=20 | >1 KU/L  N=16 | **Positive**  **N=14** | Not done N=14 |
|  |  |  | Negative N=1 | Not done N=1 **^a^** |
|  |  |  | NR N=1 | Not done N=1 **^a^** |
|  |  | <1 KU/L  N=4 | **Positive N=2** | Not done N=2 |
|  |  |  | NR N=1 | Not done N=1 **^a^** |
|  |  |  | **Negative**  **N=1** | Negative N=1 |
|  | <95% PPV  >95% NPV  N=13 | >1 KU/L  N=4 | **Positive**  **N=4** | Not done N=4 |
|  |  | <1 KU/L  N=9 | **Positive**  **N=2** | Not done N=2 |
|  |  |  | Negative N=7 | **Positive N=2**  **Negative N=3**  Not done N=2**^c^** |
|  | <95% NPV  N=6 | >1 KU/L  N=0 | N=0 | N=0 |
|  |  | <1 KU/L  N=6 | **Negative N=5** | **Negative N=5** |
|  |  |  | NR N=1 | Negative N=1 |
| Consumption  N= 0 | - | - | - | - |

**Reasons for declining OFC: ^a^**patient avoiding tree nuts and not interested in eating peanut, **^b^**results of other tests suggested peanut allergy, **^c^**previous experience of a positive OFC and fear of reacting.

**E-Figures**

**Figure E1.** Gating strategies adopted for the in house BAT on the left in grey and the Beckman Coulter BAT on the right (with modification from the manufacturer’s recommendation to include CD203c as a basophil identification marker).

CD63

CD203c


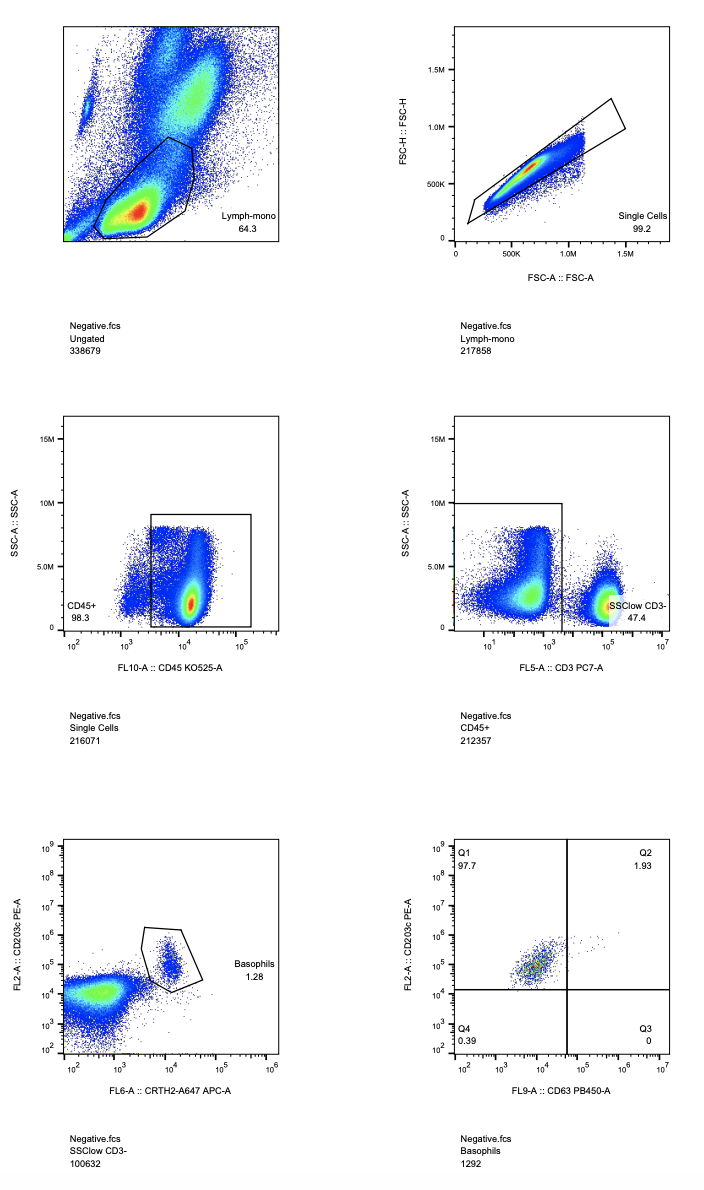


CD3

CD45


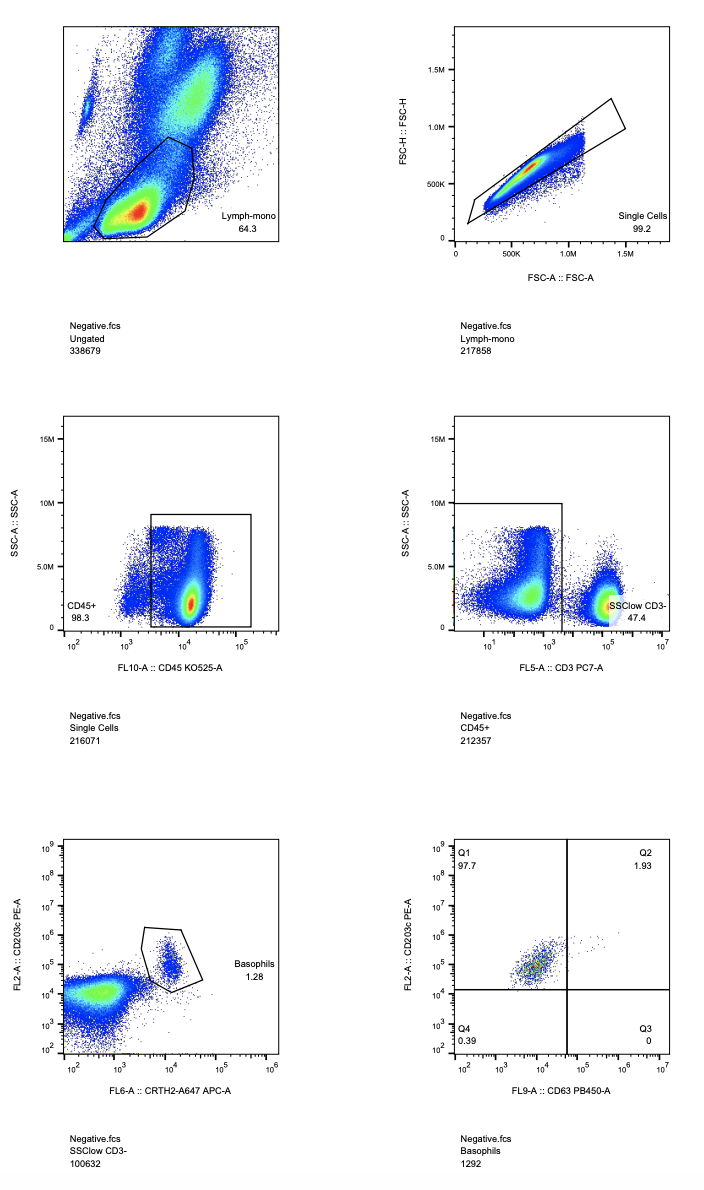


SSC

SSC

CRTH2

CD203c


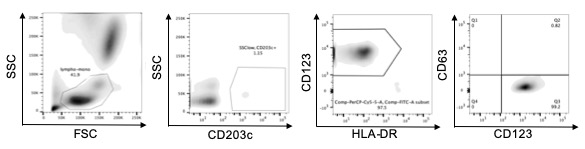

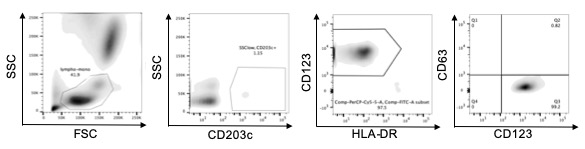


**Figure E2.** Basophil activation using the same blood sample from healthy volunteers stimulated with anti-IgE and tested by two different operators (Op1 and Op2) using two different BAT methods, in-house basophil activation test (A) and Beckman Coulter basophil activation test (B), n=10 **.**

**A.**

**B.**

**Figure E3.** Head-to-head comparison between the Beckman Coulter basophil activation test across laboratories, a research laboratory (KCL) and a diagnostic laboratory (DxLab) in terms of:

- basophil activation using CD63 (A) and CD203c (B) in a variety of stimulating conditions (RPMI alone, peanut extract 10 and 100ng/ml, anti-IgE and fMLP);
- correlation of CD63+ basophils (C) and stimulation index of CD203c (D) following stimulation with 100ng/ml of peanut extract;
- Bland-Altman plot of difference over average of basophil activation using CD63 (E) and CD203c (F).

**A B**

**C D**

**E F**

**Figure E4.** Comparison of basophil activation measured with stimulation index (SI) of CD203c using two BAT methods performed at the KCL Lab: IH, in house-BAT method and BC, Beckman Coulter method. N=66

**A.**

**B.**

**C.**

**Figure E5.** Receiver Operator Characteristic (ROC) curve for the 2 BAT methods: In-house BAT (A) and Beckman Coulter BAT (B).

**A.**

**
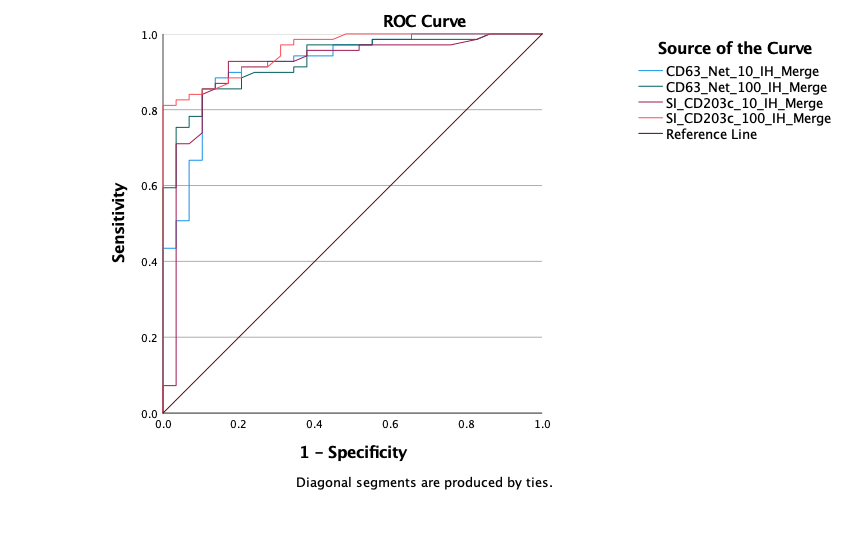
**

| Test Result Variable(s) | Area | Asymptotic 95% Confidence Interval | |
| --- | --- | --- | --- |
|  |  | Lower Bound | Upper Bound |
| CD63_Net_10_IH | .918 | .858 | .978 |
| CD63_Net_100_IH | .929 | .879 | .978 |
| SI_CD203c_10_IH | .909 | .836 | .982 |
| SI_CD203c_100_IH | .957 | .923 | .991 |

**B.**


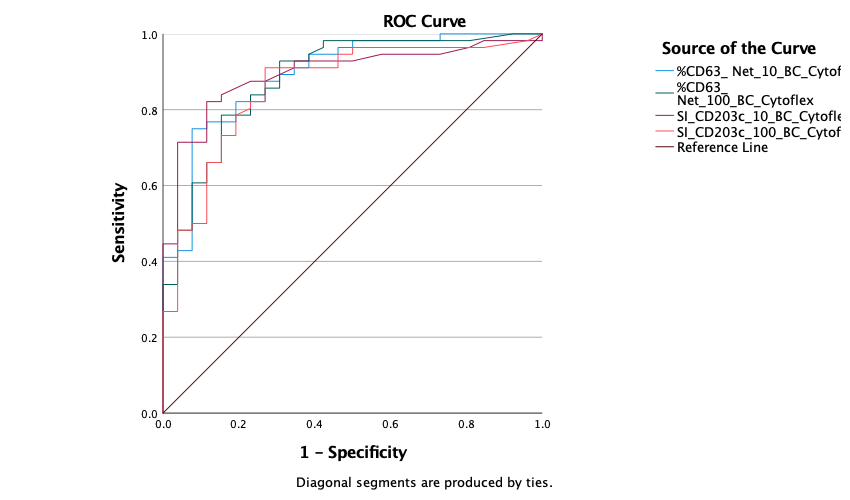


| Test Result Variable(s) | Area | Asymptotic 95% Confidence Interval | |
| --- | --- | --- | --- |
|  |  | Lower Bound | Upper Bound |
| %CD63_ Net_10_BC | .892 | .819 | .966 |
| %CD63_ Net_100_BC | .883 | .805 | .962 |
| SI_CD203c_10_BC | .895 | .824 | .965 |
| SI_CD203c_100_BC | .860 | .772 | .947 |
